# Optimizing Results of Postmastectomy Radiation Therapy Utilizing the Latissimus Dorsi Flap and Tissue Expander Technique: A Single-Center Experience

**Published:** 2017-12-20

**Authors:** Waseem Mohiuddin, Guillaume S. Chevrollier, Patrick J. Greaney, Matthew P. Jenkins, Steven E. Copit

**Affiliations:** Division of Plastic Surgery, Thomas Jefferson University Hospital, Philadelphia, Pa Presented at: The Northwest Society of Plastic Surgeons Annual Meeting, February 18-22, 2017, Big Sky, Mont.

**Keywords:** breast reconstruction, latissimus dorsi flap, postmastectomy radiation therapy, autologous reconstruction, temporizing tissue expander

## Abstract

**Objective:** Postmastectomy radiation therapy is a well-established risk factor for complications after breast reconstruction. Even if the surgeon has a suspicion that radiation therapy may be needed, it may be beneficial to place tissue expanders during the mastectomy procedure as a temporizing measure, complete radiation therapy, and then reconstruct the breast with a latissimus flap. The purpose of this study was to examine the complication rates of the latissimus dorsi flap as compared with the complication rates of implant-based reconstruction in the setting of radiation therapy. **Methods:** A 16-year retrospective chart review from 2000 to 2016 was conducted. All patients who underwent temporizing tissue expander placement for radiotherapy with subsequent latissimus flap reconstruction were included in the study. Patients who did not follow up for implant exchange were excluded from the study. **Results:** Fifty-five patients were identified with an average age of 46.0 years (range, 27-67 years) and an average body mass index of 24.2 (range, 18.9-31.9). Five patients (9.1%) developed capsular contractures amenable to surgical intervention. One patient (1.8%) developed infection of the tissue expander, requiring removal. There were no incidences of flap failure or wound dehiscence. The average follow-up after latissimus flap reconstruction was 25.3 months (range, 3.7-121.6 months). **Conclusions:** We feel that the latissimus dorsi flap after postmastectomy radiation therapy represents the preferred implant-based reconstruction option to consider when the need for postmastectomy radiation therapy is anticipated. The latissimus dorsi flap remains a safe, effective solution to postmastectomy radiation therapy that every plastic surgeon should offer.

There has been an increasing use of postmastectomy radiation therapy (PMRT) in patients with breast cancer (30%), especially in cases of large tumors (T3 or greater) or node-positive disease (N1 or greater).^1^^,^^2^ When radiation therapy for the treatment of breast cancer is expected, tissue-based breast reconstruction is generally preferred to implant-based reconstruction. This is attributed to the high rate of complications with postoperative radiation therapy after implant-based reconstruction, ranging from 20% to 70%.^3^ In cases when the need for PMRT is anticipated, breast reconstruction with autologous tissue is often delayed until the completion of the radiation therapy. This is done to avoid irradiating the flap, to optimize radiation delivery, and to avoid any additional complications that may delay adjuvant therapy.^4^^-^10 In addition, irradiation is known to cause tissue fibrosis, edema, and vasculitis, all of which can interfere with wound healing.^4^

First described by Tansini in 1896, the latissimus dorsi (LD) flap is a well-established method of breast reconstruction.^11^^,^^12^ The LD flap is a hybrid form of breast reconstruction, as augmentation with implants is usually necessary in women with moderate to large breasts.^13^ While there are numerous studies delineating the outcomes of delayed LD flap reconstruction in the setting of previous radiation therapy, delayed reconstruction may cause loss of the skin envelope and breast shape, yielding a suboptimal aesthetic result.^3^^,^^14^^-^17

For patients who are not candidates for autologous reconstruction with abdominal tissue, the use of an implant normally is required in the setting of radiation therapy. The placement of a permanent implant in the field of radiation remains a common procedure despite a well-documented excess complication profile. We believe that the use of autologous tissue with the LD flap represents for these patients a significantly better option than an implant alone and, despite its ease of use and excellent complication profile, it remains a “backup” option for many surgeons—reserved for use only after the permanent implant-based reconstruction has failed. The purpose of this study was to examine the complication rates of the temporizing tissue expander during PMRT and subsequent delayed LD flap placement. Results are compared with the complication rates of tissue expander and implant-based reconstruction with PMRT. We hope to reinforce the use of LD flap reconstruction as the “first-line” reconstruction option post–radiation therapy in those patients considered to be poor candidates for abdominally based free flap reconstruction.

## MATERIALS AND METHODS

**Figure 1:**
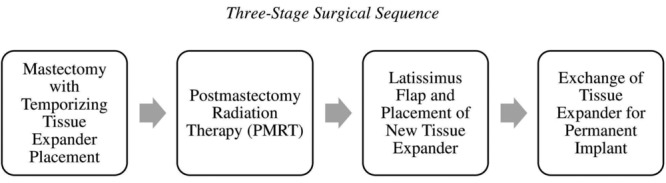
Three-stage surgical sequence employed when postmastectomy radiation therapy is anticipated.

After obtaining institutional review board approval, a retrospective chart review was conducted on all patients from 2000 to 2016 who underwent temporizing tissue expander placement for anticipated PMRT with subsequent LD flap reconstruction at our institution (Fig 1). This 3-stage method starts with the placement of a subpectoral tissue expander on the same day as the mastectomy. After radiation therapy is completed, the patient undergoes breast reconstruction with an LD flap and a new tissue expander. Finally, the tissue expander is exchanged for a permanent implant during the third stage.

All patients in the study were deemed unsuitable candidates for abdominally based free flap reconstruction because of insufficient abdominal tissue, previous abdominal surgery, or insufficient perforators demonstrated on computed tomography angiogram. Patients were excluded if they did not follow up for implant exchange. Variables examined included age, body mass index (BMI), ASA (American Society of Anesthesiologists) class, breast size, home medications, tumor type, tumor stage, laterality, timing of radiation therapy, administration of chemotherapy, comorbidities, and complications. Complications at both the donor and recipient sites were recorded, including total flap loss, hematoma, seroma, infection, capsular contracture, and wound dehiscence.

## RESULTS

**Figure 2:**
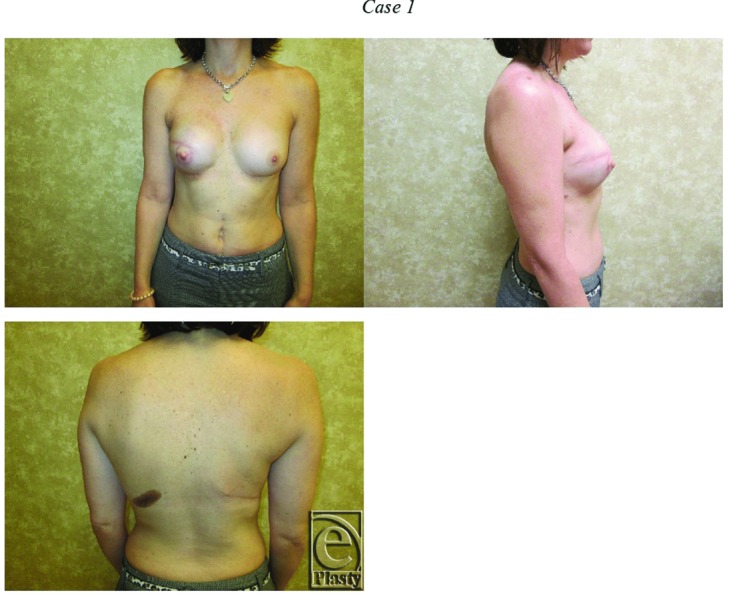
This 43-year-old woman had right breast cancer that was treated with right mastectomy, tissue expander placement, and radiation therapy. She later underwent unilateral latissimus dorsi flap placement with tissue expander. During the implant exchange procedure, she had a left augmentation for symmetry. Of note, she was a smoker and had a body mass index of 21.3.

**Figure 3:**
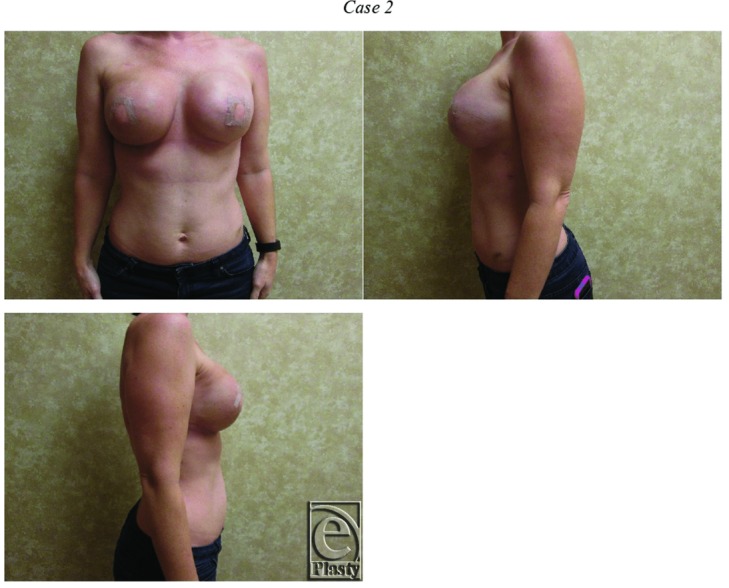
This 36-year-old woman with right breast cancer was treated with bilateral mastectomy, tissue expander placements, and radiation therapy on the right side. She underwent bilateral latissimus dorsi flap placement. Of note, she was a nonsmoker with a body mass index of 25.3.

Fifty-five patients with 55 irradiated breasts were identified, with an average age of 46.0 years (range, 27-67 years) and an average BMI of 24.2 (range, 18.9-31.9). Two patients (3.6%) were smokers, who had refrained from smoking at least 8 weeks before the latissimus flap operation. While 32 of the 55 patients underwent bilateral LD flap reconstruction, LD flaps placed in the nonirradiated side were not examined. No patients received bilateral radiation therapy. After the first-stage surgery, there were 8 tissue expander infections and 4 exposed tissue expanders, all of which necessitated tissue expander removal. In these instances, we proceeded directly to the second-stage LD flap with tissue expander. There was 1 suspected tissue expander infection that resolved with intravenous antibiotics. In addition, acellular dermal matrices were used for lower pole coverage at the time of tissue expander placement in 31 of the 55 patients—8 Alloderm (Lifecell and Acelity Corporation, Branchburg, NJ), 4 Neoform (Mentor Corporation, Santa Barbara, Calif), 13 Flex HD (Musculoskeletal Transplant Foundation, Edison, NJ), and 6 Allomax (Bard, Warwick, RI). Typical postoperative results are shown in Figures 2 and 3.

The most common complication was donor site seroma, which occurred in 31 patients (56.4%). These were treated with serial aspirations in the office. No seromas required operative intervention. In addition, 1 breast seroma and 1 breast hematoma occurred (1.8%). Five patients (9.1%) developed capsular contractures of grade II or higher amenable to surgical intervention. One patient (1.8%) was found to have an infected permanent implant, which necessitated operative replacement (Table 1). There were no incidences of flap failure or wound dehiscence. The mean follow-up after latissimus flap reconstruction was 25.3 months (range, 3.7-121.6 months), and the mean time to implant exchange was 5.4 months (range, 2.1-13.1 months).

## DISCUSSION

**Figure 4:**
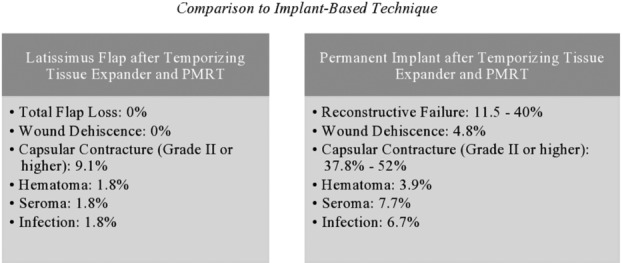
Outcomes of the latissimus flap after temporizing tissue expander and PMRT compared with the outcomes of temporizing tissue expander with PMRT and subsequent implant exchange. PMRT indicates postmastectomy radiation therapy.

It is well established that in patients receiving PMRT, outcomes of autologous reconstruction are superior to implant-based reconstruction.^18^ In a series of 104 patients who underwent radiation therapy for the tissue expander with implant exchange, Santosa et al^19^ reported a 30.8% complication rate and an 11.5% incidence rate of reconstructive failure. Similarly, Cordeiro et al^20^ demonstrated a reconstructive failure rate of 18.1% and 32.0% at 6 years when applying Kaplan-Meier analysis. In a prospective study of 50 patients who received radiotherapy during the expansion phase, Nava et al^21^ also found an unacceptably high rate of reconstructive failure of 40%. These studies were examined together to compare the complication rates with those of the current study population (Fig 4).

To our knowledge, this is the only study looking specifically at the outcomes of this 3-stage method. In 2014, Clemens et al^22^ published a study of a cohort of patients who underwent “delayed-immediate reconstruction,” a technique that also involves the placement of a temporizing tissue expander for PMRT. The use of the tissue expander prior to the radiation phase helps preserve the shape and thickness of the breast skin flaps and the dimensions of the breast envelope while maintaining landmarks such as the inframammary fold.^23^ In addition, it should be noted that the delayed-immediate reconstruction by Clemens et al^22^ places the LD flap with a permanent implant, so no final exchange procedure is necessary. In their 64-patient series of delayed-immediate LD flaps, they reported rates of capsular contracture, infection, and breast seroma of 4.7%, 8.3%, and 8.9%, respectively. These results compare well with our results of 9.1%, 1.8%, and 1.8%, respectively.^14^^,^^22^

One potential criticism of this technique is that the complications resulting from immediate placement of tissue expanders may delay oncologic care. However, this seems to be an insignificant phenomenon.^18^ Kronowitz et al^24^ conducted a study comparing delayed-immediate reconstruction with standard delayed reconstruction. They found no significant difference in 3-year recurrence-free survival rates.^24^ Even in patients who underwent a procedure as extensive as free flap reconstruction, Crisera et al^25^ found that the maximal delay in administration of postoperative chemotherapy was only 3 weeks. This is most likely oncologically insignificant, given the results of Buzdar et al,^26^ who found no difference in disease-free survival when chemotherapy was delayed less than or greater than 10 weeks postoperatively. In addition, our expander loss rate of 22% compares well with that of Nava et al,^21^ who made no mention of using an acellular dermal matrix. While publications looking specifically at outcomes of radiation therapy to a tissue expander and acellular dermal matrix are rare, Ortiz^27^ reports a similar 21.4% expander loss rate. Bearing this in mind in addition to the benefits of immediate tissue expansion—preservation of the inframammary fold, improved skin envelope preservation, increased skin, improved esthetics, and the positive psychological impact of allowing the patient to wake with a breast mound,^22^^,^^23^ we feel that the use of immediate tissue expansion is justified.

Rates of donor site seroma for the LD flap are quoted from 5% to 80%.^28^ Although a donor site seroma rate of 56.4% in our cohort is not ideal, it should be noted that all seromas were managed with serial aspirations and that no seroma required operative intervention. While performing bilateral latissimus flap reconstruction may result in more frequent complications than unilateral cases, bilateral LD flap reconstruction employed in the appropriate patient produces improved cosmetic outcome and improved symmetry. On the basis of the results achieved, we maintain that the LD flap remains a viable choice in reconstructing the irradiated breast.

There has been an increasing interest in the use of fat grafting for breast reconstruction.^29^ While we agree that fat grafting has shown promising results, it has some limitations in our patient population. Primarily, only 3 of our 55 patients underwent a nipple-sparing mastectomy. In all other cases, a significant amount of skin was resected, which necessitated skin to be taken from a donor site. In addition, the skin that remained showed significant radiation damage that benefited more from reconstruction with nonirradiated skin and muscle than large-volume fat grafting. While we recognize that fat grafting and acellular dermal matrices are useful adjuncts, we find that there is minimal evidence demonstrating their superiority to the LD flap in the setting of radiation therapy. After reconstruction with the LD flap, any contour abnormalities that remain can be later corrected with fat grafting.

Initial reports of the latissimus flap for breast reconstruction described its use with permanent implants. However, this technique resulted in an unacceptably high rate of capsular contracture (21%-75%), which may explain why it has been less popular. The 2-stage method of tissue expander placement at the time of mastectomy and LD flap, followed by implant exchange, has resulted in a lower capsular contracture rate of less than 10%. This may be explained by the expander opposing contractile forces of the early wound while weakening the contracture before permanent implant placement. This softer capsule could also be released or adjusted at the time of implant exchange to provide a better pocket for the implant.^30^^,^^31^ For these reasons, we prefer to place a tissue expander at the time of latissimus flap placement despite the need for another surgery.

The limitations of this study stem mainly from its retrospective nature and inability to randomize patients to an implant-only group for comparison. Furthermore, despite the relatively long mean follow-up period of 25.3 months, a more extended follow-up with more patients may have captured more complications. While all patients achieved satisfactory aesthetic outcomes, a more objective way of grading the cosmetic results would have further strengthened this study.

## CONCLUSION

We feel that the LD flap after PMRT represents the preferred implant-based reconstruction option to consider when the need for PMRT is anticipated. When compared with the reconstructive failure rate of irradiated tissue expanders with implant exchange, the temporizing tissue expander with subsequent autologous tissue coverage yields favorable outcomes. Especially when abdominally based free flap procedures are not feasible or not desired by the patient, the LD flap should be increasingly utilized by plastic surgeons to reconstruct the irradiated breast. Given its reliability and low short- and long-term complication rates, it remains a perplexing question as to why many surgeons continue to place a permanent implant post–radiation therapy without autologous tissue. For those patients who are not candidates for abdominally based free flap reconstruction or for those surgeons without microsurgery capability, the LD flap remains a safe, effective solution to PMRT that every plastic surgeon should offer.

## Figures and Tables

**Table 1:** Recipient site complications in the study population

Complication	Number of Patients (%)
Total flap loss	0 (0)
Wound dehiscence	0 (0)
Capsular contracture (grade II or higher)	5 (9.1)
Hematoma	1 (1.8)
Seroma	1 (1.8)
Infection of second tissue expander	1 (1.8)
Failure of first tissue expander	12 (21.8)
